# Searching Remote Homology with Spectral Clustering with Symmetry in Neighborhood Cluster Kernels

**DOI:** 10.1371/journal.pone.0046468

**Published:** 2013-02-15

**Authors:** Ujjwal Maulik, Anasua Sarkar

**Affiliations:** 1 Department of Computer Science and Engineering, Jadavpur University, Kolkata, West Bengal, India; 2 Theoretical Bioinformatics, German Cancer Research Center (dkfz), Heidelberg, Germany; 3 LaBRI, University Bordeaux 1, Talence, France; American University in Cairo, Egypt

## Abstract

Remote homology detection among proteins utilizing only the unlabelled sequences is a central problem in comparative genomics. The existing cluster kernel methods based on neighborhoods and profiles and the Markov clustering algorithms are currently the most popular methods for protein family recognition. The deviation from random walks with inflation or dependency on hard threshold in similarity measure in those methods requires an enhancement for homology detection among multi-domain proteins. We propose to combine spectral clustering with neighborhood kernels in Markov similarity for enhancing sensitivity in detecting homology independent of “recent” paralogs. The spectral clustering approach with new combined local alignment kernels more effectively exploits the unsupervised protein sequences globally reducing inter-cluster walks. When combined with the corrections based on modified symmetry based proximity norm deemphasizing outliers, the technique proposed in this article outperforms other state-of-the-art cluster kernels among all twelve implemented kernels. The comparison with the state-of-the-art string and mismatch kernels also show the superior performance scores provided by the proposed kernels. Similar performance improvement also is found over an existing large dataset. Therefore the proposed spectral clustering framework over combined local alignment kernels with modified symmetry based correction achieves superior performance for unsupervised remote homolog detection even in multi-domain and promiscuous domain proteins from Genolevures database families with better biological relevance. Source code available upon request. Contact: sarkar@labri.fr.

## Introduction

The remote homology detection from available protein sequences is one fundamental problem in comparative genomics. With higher sequence similarity, several panoply of methods can detect homologs accurately. However detecting remote homologs with subtle sequence similarity still remains a challenging problem.

In general, there are three categories of methods to solve this problem – simple approaches based on sequence similarity like BLAST or Smith-Waterman [1], [2], generative model approaches like HMMs (Hidden Markov Models) [3], [4] and discriminative classifier methods like SVMs (Support Vector Machines) [5]–[7]. Historically, the probabilistic profiles (PSSMs) method (PSI-BLAST) [8] exhibits superior performances for remote homology.

Recently, the discriminative kernel methods with SVMs like mismatch string kernels [6], [9], string alignment kernels [10], profile-based direct kernels [11] – exhibited better homology detection. These methods require extensive annotated proteins for training to yield good performances. The protein-structure kernel on MAMMOTH score in [12] and the combined approach of sequence and secondary-structure similarity scores in [13] also proved to be efficient. Incorporating incremental-kernel [14], multi-instance kernel [13] or gapped Markov-feature pairs [15] are the recent approaches for homology detection.

To compute the sequence distances, some groups utilized Connected Component Analysis(CCA) [16] on fully-connected graphs like GeneRAGE [17]. To improve them, Markov cluster algorithm(MCL) [18] utilizes random walks on Markov transition matrix to analyse the emergence of clusters in the graph, which encodes this matrix. The most successful methods for homology detection utilizing MCL algorithms [18] are OrthoMCL [19] and TribeMCL [20], which bias the random walks with ‘inflation’ parameter to promote the cluster emergence. Earlier non-kernel approach of [21] significantly utilize spectral clustering on protein sequences.

The semi-supervised protein clustering achieved efficiency earlier, introducing the neighborhood vector over profiles in cluster kernels by [22], [23]. The combined kernel approach using bagging-method over mismatch-string kernels [22] utilized the strength of combined clustering for remote homology. The protein-function prediction with kernels on Yeast genomes [24], introduced one kernel matrix for combining heterogeneous data.

Symmetry is an inherent feature to enhance recognition and reconstruction of shapes and objects. It reflects to be powerful for recognizing homolog protein clusters in kernel space. In [25] a symmetry based distance measure is proposed. Yet it fails to detect clusters with inherent symmetry relative to some intermediate point. Subsequently, the distance norm is corrected in [26] leading to a modified proximity norm, which is able to handle overlapping symmetrical clusters with multiclass points.

In this work, at first we develop new valid Mercer kernels based on similarities explicitly in local alignment methods like BLAST and PSI-BLAST. We present two positive semi-definitive local-alignment kernels based on the singular-value decompositions of respectively MCL similarity scoring and position-specific scoring matrices (profiles). The Markov cluster similarity kernel further with the neighborhood feature vectors is enhanced. Furthermore incorporating the mismatches with profiles the diagonal dominance issue problem is reduced. This enables more accurate detection of remote homologs boosted by similarity deemphasizing multi-domain proteins. To reduce promiscuous domain problems, we further incorporate the spectral clustering approach over kernel matrices to alleviate inter-cluster edges implicitly selecting the leading eigenvectors from ‘global’ distances without using any hard-threshold. Finally, we introduce the modified-symmetry based correction over the homolog distributions in Hilbert space. This reduces number of singletons (represented as outliers) and classifies multi-domain proteins into more biologically-significant clusters with closest nearest-neighbor homologs from different domains. Contradicting with earlier discriminative approaches, this approach detects remote homology among unlabelled multi-domain proteins without any prior annotation. Local-alignment kernels or Markov similarities are combined cascadingly with neighborhoods in spectral clustering, which are further enhanced by modified-symmetry based correction.

We experiment all our kernel frameworks over the multi-domain proteins from Genolevures Yeast database [27]. The performance of our combined spectral kernels with modified symmetry are compared to other state-of-the-art combined cluster kernel methods. The experimental outcomes also demonstrate the superiority of introducing modified-symmetry over kernel space with spectral clustering to detect remote homologs more accurately even for multi-domain and promiscuous domain proteins. Moreover statistical and quantitative performance evaluations with five validity measures to demonstrate the significance of our proposed approaches are also performed. We also study the comparative results over our chosen dataset provided by the already-existing string [28] and mismatch [22] kernels with our proposed kernels. To experiment over the large datasets, we compare the clustering solutions of our proposed kernels with those of the already-existing string [28] and mismatch [22] kernels over the sequences of target 54 families from SCOP version 1.59 [22]. The scores provided by all algorithms also show the superiority of our proposed kernels with higher values.

## Materials and Methods

### Background

In this section, we briefly describe existing state-of-the-art cluster kernel methods for remote homolog proteins detection and the modified symmetry based distance measure for clustering.

#### Spectral clustering

In semisupervised learning, [23] introduced cluster kernels modifying the eigenspectrum of a kernel matrix. The spectral clustering kernel boils down to be the spectral graph partitioning into the sub-space of the 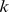 largest eigenvectors of a normalized affinity/kernel matrix [29]. Let us assume an undirected graph 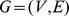 with vertices 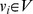, for 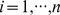 and edges 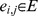 with non-negative weights 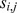 expressing the similarity between vertices 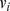 and 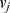. Then the eigenvectors 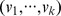 are computed as 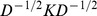, where 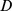 is a diagonal matrix computed as 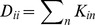, where 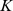 is the RBF-kernel interpreted as a transition matrix of random walk on the graph. The spectral clustering approach produced qualified clusters from protein sequences earlier [21], [23] following the work of Weiss [29] and Mealia and Shi [30] to simultaneously analyse 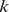 eigenvectors before normalizing.

#### Neighborhood mismatch kernel

To project the selection of closely related neighbor sequences through evolution from PSI-BLAST profiles in mismatch kernel, [22] defined a neighborhood kernel over the feature representation 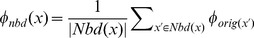 as shown below:
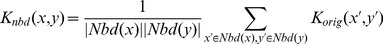
(1)where 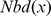 denotes a neigborhood for sequence 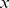 over a sequence set 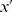 with E-value less than a fixed threshold in PSI-BLAST/BLASTP search. As they proved the neighborhood averaged vector 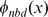 stays within the convex hull of all vectors in neighborhood [22], this kernel boosts up the protein classification performance.

#### Modified symmetry based distance measure

Among the different distance measures for clustering like Euclidean, Pearson correlation or Spearman distance, none can detect symmetrical overlapping clusters. Su and Chou [25] proposed a symmetry based distance measure 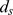 between a pattern 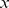 and a reference centroid 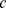 as follows:
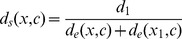
(2)where 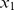 is the symmetrical point of 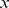 with respect to 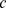 and 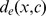 and 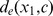 are Euclidean distances respectively between 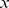 and 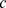 and between 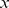 and 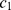. If 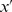 represents the first nearest-neighbor of 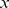 and is computed as 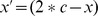, then 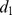 represents Euclidean distance of 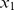 and 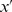. To improve the effect of this symmetry-based distance norm even for inter-symmetrical clusters, Chou et al [26] proposed a modified measure 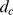 as defined below:



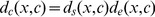
(3)Therefore to detect compact symmetrical overlapping clusters we incorporate the modified-symmetry based distance measure [26]. This improves the biological significance of homology detection reducing outliers, as we discuss later.

### Data

The Genolevures database explores nine complete genomes (*Candida glabrata, Eremothecium gossipii, Kluyveromyces Lactis, Yarrowlo lipotytica, Zygosaccharomyces rouxii, Saccharomyces kluyveri, Kluyveromyces thermotolerans, Debaryomyces hansenii, Saccharomyces cerevisiae*) [31], [27] from the class of Hemiascomycete yeasts. The non-redundant protein-family database was generated by progressively taking protein-coding gene-sequences following the family structures of Genolevures Release 3 candidate 3 data (2008-09-24) [32]–[33]. We use 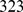 sequences as unlabelled data from 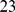 Multiple choice families 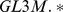 which are complicated families like polyproteins and repeat-domains. Therefore proper homology detection among them is suitable for our remote homology experiments. We use the Genolevures Release-3 candidate-3 [32] family structure as the true-clusters for ROC analysis. Finally we utilize 1000 sequences of the target 54 families from SCOP version 1.59 which it was experimented earlier in [22] for testing the performances of our proposed kernels over the large datasets. This dataset contains the kernel matrices generated from BLAST, PSI-BLAST and Spectrum mismatch kernels following the method of [22].

## Methods

To explore remotely detected homologs even for multi-domain and promiscuous domain proteins, we define twelve simple and combined alignment cluster kernels in this section and evaluate them with spectral clustering.

### Local alignment-based kernels

The local alignment kernel developed in [10] based on SW (Smith-Waterman) scores. They measured the pair-wise sequence similarity by summing up local alignment scores with sequence gaps. They use a convolution of kernels with a point wise limit to the Mercer kernels. The probabilistic profiles of logarithmic E-values generated by local alignment methods like BLASTP or PSI-BLAST are recently used for kernel generation instead of sequence encoding itself for protein classification [21]. However collecting these E-values for a pair of sequences into a matrix does not satisfy symmetric property in the alignment scores. The average interpretation of log10 of E-values between two sequences produces a symmetric kernel solving this problem in MCL algorithm [20]. This symmetric matrix is represented as a connection graph with weighted edges between proteins, which are searched iteratively for probabilities of protein transitions and matrix inflations by scaling the Hadamard power of the matrix.

However utilizing the HSP (high-scoring segment pair) score of BLASTP results directly resembles the functionality of mismatch string kernel [6] to some extent. Therefore instead of using the E-values as in earlier works for kernel formation, we utilize the BLASTP HSP score within the threshold cut-off to compute the kernel matrix which also satisfies the biological relevance of searching out homologous sequences. We define this kernel as kernel (**I**).

#### Position specific scoring kernel

To explore the statistically significant alignments produced by BLASTP with the position-specific score matrix (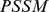), PSI-BLAST generates a score to the iterated gapped multiple alignment over a set of sequences [8]. We treat the PSI-BLAST score directly for generating the kernel matrix computation, as it represents the similarity of homologous sequences in descending order more accurately than BLASTP [1], [34]. Unfortunately the matrix formed directly from PSI-BLAST scores between pair of sequences is not positive semidefinitive in nature, as all-vs-all PSI-BLAST scores are not symmetric for a pair of sequences. However if 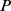 is the PSI-BLAST similarity score matrix, then 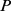 is symmetric with singular value decomposition 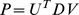 where 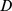 is the diagonal matrix 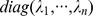 with singular value entries 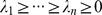. Therefore we define the PSI-BLAST kernel by
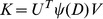
(4)where 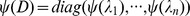 and 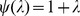 if 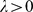, and 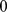 otherwise. We normalize the kernel with unit sphere projection via, 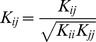. We identify this kernel as kernel (**II**). A related protein structure kernel, based on MAMMOTH score [12] previously yielded good performance in classifying proteins.

#### Markov cluster similarity scoring kernel

The Markov Cluster algorithm(MCL – http://micans.org/mcl/) [18] is a fast and reliable approach for complicated domain structures [20], which simulates random walks on a graph to detect the transition probabilities among its edges using Markov matrices. Several existing methods including TribeMCL [20] and OrthoMCL [19] apply the MCL algorithm to detect protein clusters which consists of multi-species orthologs or recent paralogs. The scoring matrix used for MCL clustering in OrthoMCL algorithm is initially computed as the average 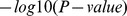 from pairwise WU-BLASTP similarities. These weights are then normalized dividing the averaged edge weights 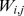 of all ortholog pairs of two species 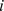 and 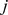 by average weight 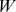 of all multi-species ortholog and “recent” paralog pairs [19]. This minimizes the impact of “recent” paralogs in cross-species ortholog clusters. Therefore this normalized score emphasizes the remote homologs better than the BLASTP scores and also reduces the impact of “recent” paralogs in classification. We generate another kernel matrix using this score, which solves the diagonal dominance issue for 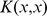 to be orders of magnitudes larger than 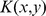, by assigning arbitrary values to 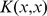. To satisfy the positive semidefinitive property in this kernel, we utilize the neighbors and the profiles information to transform this matrix.

#### Neighborhood similarity kernel

We incorporate the neighborhood probabilistic representation of each input sequence over the above explained MCL similarity scores, following earlier neighborhood mismatch kernel [22]. Initially we compute the neighborhood feature vector over the MCL scores and then generate neighborhood similarity matrix in equation 1. However to satisfy the positive semidefinitive property of our kernel we compute the singular value decomposition of this matrix. We normalize the generated kernel to the [0,1] interval. We identify our OrthoMCL Neighborhood Mismatch 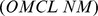 kernel as kernel (**III**).

#### Mismatch profile kernel

To construct the kernel based on profile information, we generate a variant kernel with MCL similarity and PSI-BLAST profile-based scores. Following the profile mismatch kernel based on spectrum kernel [23], we develop our kernel using the probabilistic profiles of sequences over the neighborhood of the Markov cluster similarity kernel. The singular value decomposition over our feature vector with the [0,1] interval normalization generates our new kernel with semi-definitive property. We identify our OrthoMCL Mismatch Profile 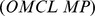 kernel as kernel (**IV**).

### Combined spectral kernel clustering

The position specific scoring kernels are based on the singular value decompositions and therefore, are Mercer's kernels. Again the neighborhood similarity kernel and mismatch profile kernel are also proved to be Mercer kernels. We define the kernels combining 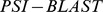 with 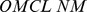 and 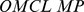 kernels as kernels (**V, VII**) and similarly the combined 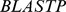 kernels with 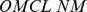 and 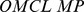 kernels as kernels (**IV, VI**). Therefore our combined local alignment kernels (**V, VI, VII, VIII**), which are the tensor products 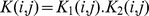 of those simple alignment and modified Markov cluster similarity kernels are also valid Mercer's kernels [35]–[37].

For unsupervised classification, we apply the spectral clustering method directly to the combined local alignment cluster kernel matrices without using a transductive setting like in [22]. [12] established the well-clustered approach of the spectral clustering over protein sequences. However this random walk based graph partitioning method solves the problem to identify the tightly coupled clusters, and cut the inter-cluster edges. Thus explicitly removing the promiscuous domain problem.

This algorithm also constructs the Markov transition matrix as used in Markov Clustering algorithm (MCL) [20], but differs in the analysis of the perturbation to the stationary distribution following a Markovian relaxation process [12] to utilize the eigenvectors corresponding to the leading eigenvalues of the matrix. As this method does not need to modify the random walks with a relaxation parameter called ‘inflation’ in OrthoMCL [19] and TribeMCL [20], it outperforms those methods in the accuracy of the result clusters with respect to the true classifications.

### Modified symmetry in kernel space

The modified-symmetry based distance measure 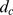 [26], as defined in equation 3 considers the nearest neighbor of symmetrical points among clusters to compute distances. The distance of a point and its nearest neighbor in the Hilbert space produces significant higher values for the case of outliers. Therefore scaling it with the euclidean distance between the point and the centroid distinguishes outliers with much higher values. Correcting clusters with lower modified symmetry norm (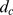) value imposes compact clusters reducing outliers over kernel space. We can define the modified symmetry based reassignment of a point 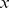 to cluster 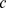 as:
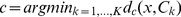
(5)where 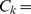 Centroid of 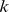
h cluster and 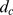 as defined in Eq 3.

Furthermore to prove the non-negative definiteness in spectral kernel with modified symmetry, for arbitrary 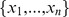, we can show that:
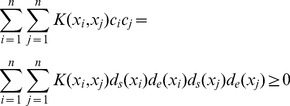
(6)where 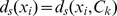 and 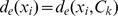 are related to 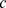 in Eq 5 using Equation 3 and are always 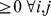.

Therefore the spectral kernel matrix with modified symmetry norms is itself positive semidefinitive in nature. Alternatively, let 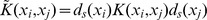, where 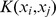 is a positive semidefinite spectral kernel. Then for arbitrary 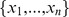 and if 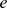 represents 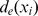 and 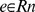, then we obtain:
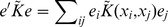


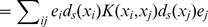


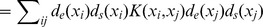


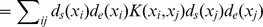


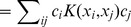


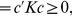
(7)where 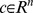 and any 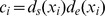 following Eq 5. Thefore 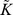 is a valid kernel function.

Accordingly, we correct the combined spectral kernel results with modified symmetry with reallocating proteins to a cluster with its optimal modified symmetry distance norm less than the pre-defined threshold 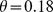 [25]. With respect to the original “true” clusters, this yields to create good overlapping symmetrical clusters, which are more relevant to homology detection as discussed in Section0. We define the spectral clustering solutions after modified symmetry based redistribution for the combined BLASTP kernel with OMCL NM and OMCL MP kernels as respectively kernels (**IX, XI**) and combined PSI-BLAST kernel with OMCL NM and OMCL MP kernels as respectively kernels (**X, XII**).

## Results

In this section the framework for the experiments and comparative results of all local alignment kernels and combined spectral kernels after modified symmetry based correction are described. The comparative study of the clustering solutions of the existing string [28] and mismatch [22] kernels are also included in this section. Similarly we perform the experiments over one large dataset also to evaluate performances of all the kernel algorithms.

### Evaluation framework

Several frameworks have been implemented for demonstatating the performance of twelve different kernels proposed in this article. The 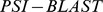 [8], [34] iterations with composition based statistics [38] are performed on a Cluster with 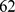 Opteron nodes [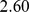 GHz, 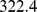 GFLOPs] using 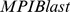 and the command-line program 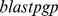. We implement OrthoMCL version 2.0 [19] for our experiments. All the kernels are generated in Matlab v7.10 (R2010a) 64-bit. The normalized spectrum kernel with sub-sequence/string length  = 4 settings in the Kernel-based Machine Learning Lab 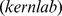 package [39] in 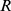 [40] from CRAN is used. This is utilized for spectral clustering [29] over all our local alignment and combined kernel matrices. The spectral clustering results of all methods are evaluated using the receiver operating characteristic (ROC) score, commonly called Area Under ROC Curve (AUC) and the ROC-50, which is the ROC score or AUC computed only up to the first 50 false positives. For the 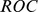 [41] analysis of the kernel matrices, 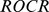 packages [42] have been used. Finally the 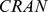 statistical package 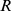 [40] with 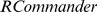 library [43] have been used for Wilcoxon signed rank test. The modified symmetry based clustering approach using MPICH has been implemented. We utilize the existing string kernel of LIBSVM [28] software for comparing its results with our kernel clustering results. We also experiment over our chosen dataset with the pre-existing spectrum mismatch [22] kernels on SVM. To verify the performances over a large dataset, we execute all our proposed as-well-as those already-existing kernels over the chosen 54 target families from SCOP version 1.59 [22] from literature as mentioned in Data section. We also utilize the linear kernel with SVM of SPIDER [44] framework in MATLAB to obtain the comparative results.

### Performance of local alignment-based spectral kernels

**Figure 1:**
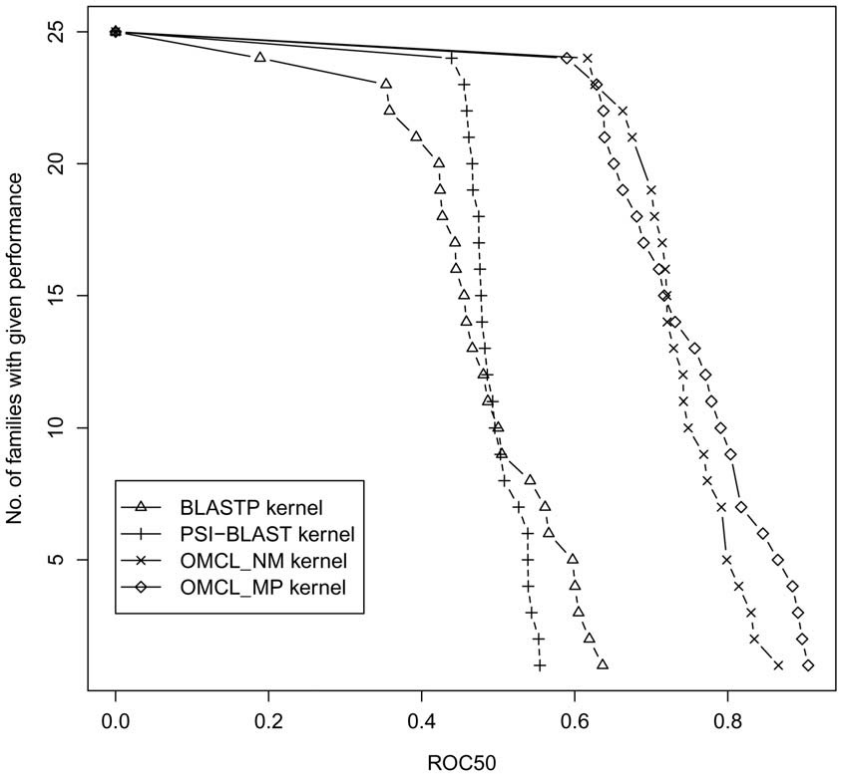
Comparison of ROC50 score distribution for different local alignment based kernels.

Table 1 summarizes the performance achieved by the local alignment based kernels for family-level classification implemented with spectral clustering. We measure the performance of BLASTP kernel(**I**), PSI-BLAST kernel(**II**), OrthoMCL Neighborhood Mismatch 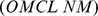 kernel(**III**) and OrthoMCL Mismatch Profile 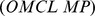 kernel(**IV**) to classify the multi-domain protein families of our dataset with mean ROC and mean ROC50 scores. These results show that 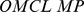 kernel(**IV**) performs best over all other methods indicating the influence of profiles in homolog detection. All the modified local alignment kernels outperforms simple score based kernels in this experiment. As an illustration, the distribution of ROC50 scores for all local alignment-based kernels is shown in Figure 1. The number of families whose ROC50 scores are greater than a given threshold in the range [0,1] are shown in Figure 1. All modified kernels from OMCL scores, namely 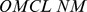
(**III**), 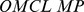
(**IV**) kernels retrieve approximately two times more ROC50 scores than the two simple score based BLASTP(**I**) and PSI-BLAST(**II**) kernels for similar number of families.

**Table 1:** ROC, ROC50 averaged over 23 families for different local alignment based kernels.

ID	Kernel	Mean ROC50	Mean ROC
I	BLASTP kernel	0.481	0.836
II	PSI-BLAST kernel	0.495	0.939
III	OMCL NM kernel	0.741	0.949
IV	OMCL MP kernel	0.756	0.960

OMCL NM = OrthoMCL Neighborhood Mismatch kernel, OMCL MP = OrthoMCL Mismatch Profile kernel.

### Performance of combined spectral kernels

**Figure 2:**
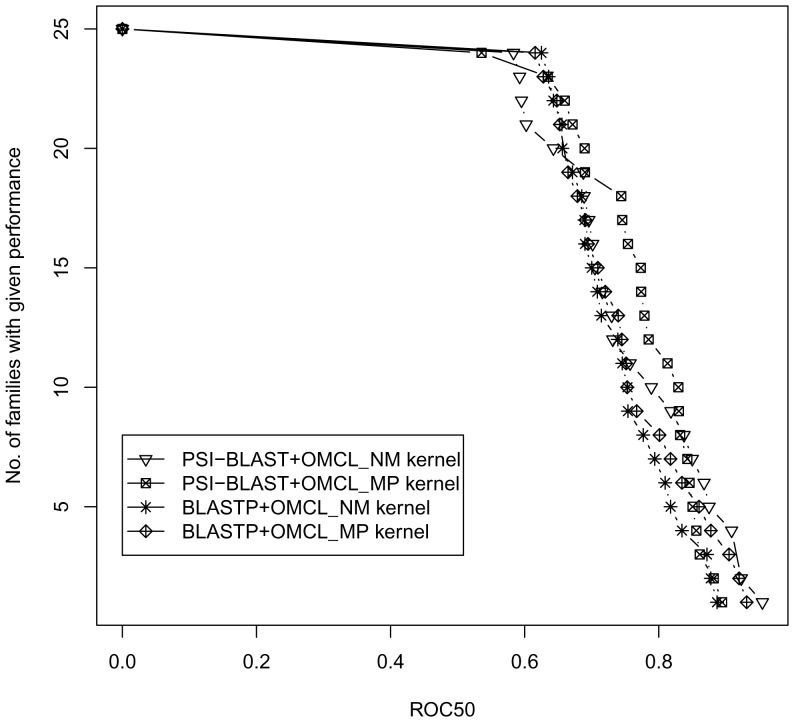
Comparison of ROC50 score distribution for different combined spectral kernels.

In order to investigate the performance of our spectral kernels over simple alignment kernels, we combine all modified local alignment kernels using normal product. Combining 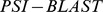 with 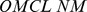
(**VI**) and 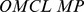 (**VIII**) kernels provide respectively ROC values 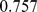 and 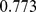 in Table 2, which is superior to the values 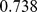 and 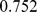 obtained by combining 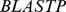 kernel respectively with 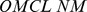 (**V**) and 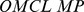 (**VIsI**) kernels. 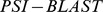 with 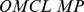 kernel (**VIII**) outperforms all other methods with the highest ROC50 score of 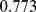. Figure 2 illustrates the combined kernel performances of ROC50 distribution for the unlabelled protein family classification. The basic BLASTP (**I**) and PSI-BLAST (**II**) kernels cannot successfully perform in the absence of sufficient positive training data for a huge unlabelled protein database [7]. Therefore combining local alignment kernels may provide improvement for unsupervised protein family classification. As shown in Figure 2 both 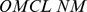 (**VI**) and 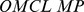 (**VIII**) kernels combined with the proposed 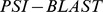 kernel (**II**) consistently show superior performance while significantly outperforms other combined kernels.

**Table 2:** ROC, ROC50 averaged over 23 families for different combined spectral kernels.

ID	Kernel	Mean ROC50	Mean ROC
V	BLASTP + OMCL NM kernel	0.738	0.942
VI	PSI-BLAST + OMCL NM kernel	0.757	0.945
VII	BLASTP + OMCL MP kernel	0.752	0.964
VIII	PSI-BLAST + OMCL MP kernel	0.773	0.961

OMCL NM = OrthoMCL Neighborhood Mismatch kernel, OMCL MP = OrthoMCL Mismatch Profile kernel.

### Modified symmetry in protein classification

In the unsupervised setting of homolog detection, the simple score based kernels do not show very strong performance in comparison with the combined modified spectral alignment kernels. Incorporation of the modified symmetry based cluster correction imporves the performance further (see Table 3) for unlabelled data. In comparison with the ROC and ROC50 scores shown in Table 2, all combined spectral kernels show better performance after modified symmetry-based enhancement in detecting homologs. The most striking observation from this result is that the major impact of modified proximity norm 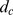 in ROC50 scores of 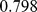 and 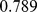 for two combined 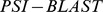 spectral kernels (**X, XII**).

**Table 3:** ROC, ROC50 averaged over 23 families for different combined spectral kernels after modified symmetry based correction.

ID	Kernel	Mean ROC50	Mean ROC
IX	BLASTP + OMCL NM kernel + Modsym	0.742	0.946
X	PSI-BLAST + OMCL NM kernel + Modsym	0.798	0.962
XI	BLASTP + OMCL MP kernel + Modsym	0.768	0.964
XII	PSI-BLAST + OMCL MP kernel + Modsym	0.789	0.969

OMCL NM  =  OrthoMCL Neighborhood Mismatch kernel, OMCL MP  =  OrthoMCL Mismatch Profile kernel.

**Figure 3:**
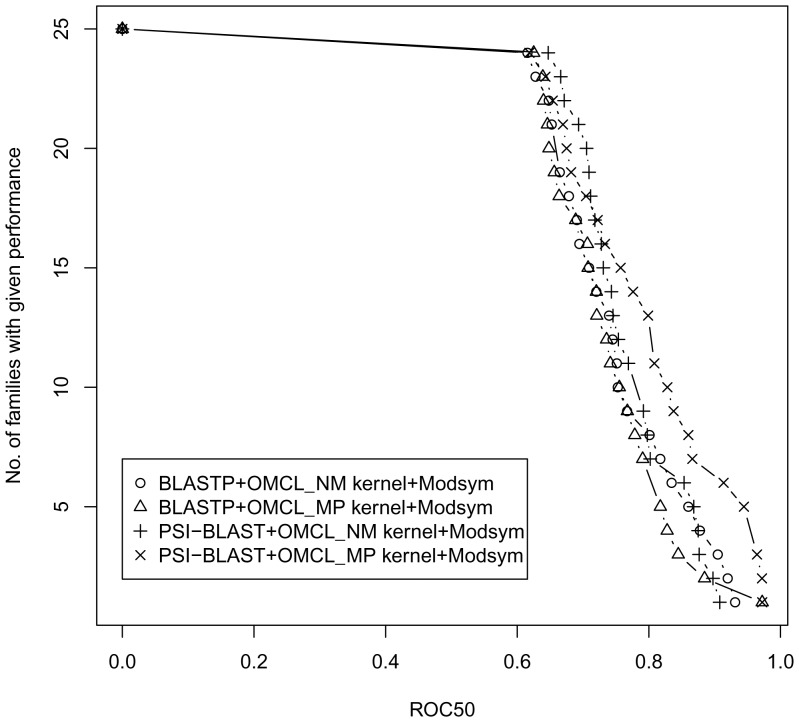
Comparison of ROC50 score distribution for different combined spectral kernels after modified symmetry based enhancement.

Figure 3 shows the ROC50 distributions for all combined 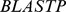 and 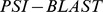 kernels after modified symmetry based corrections (**IX, X, XI, XII**). These results show that 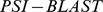 kernel combined with 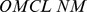 and 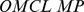 kernels after modified symmetry based redistribution (**X, XII**), consistently outperform other combined kernels with higher ROC50 values.

**Figure 4:**
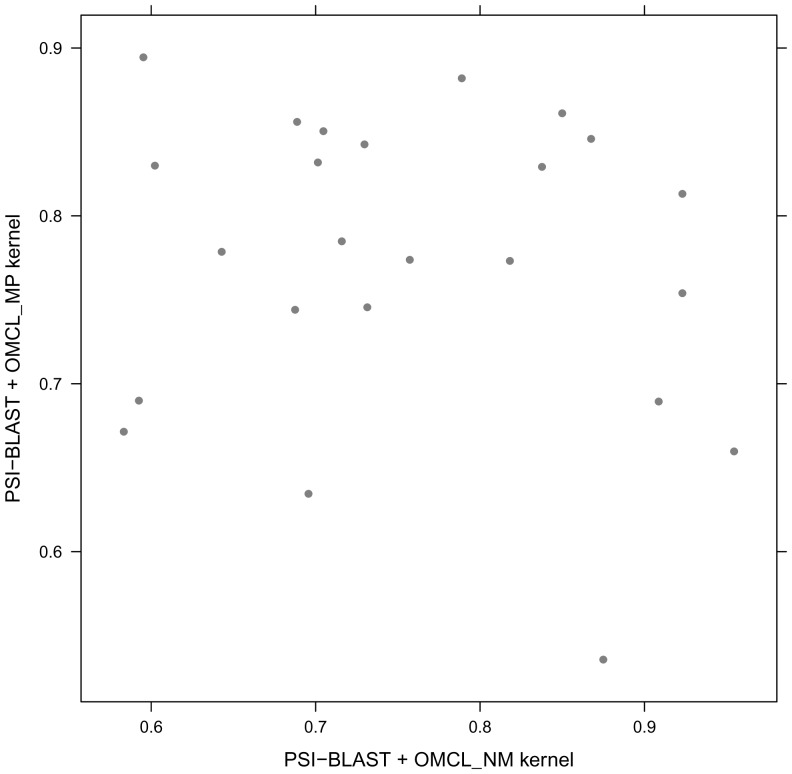
Family-by-family comparison of PSI-BLAST OMCL NM and PSI-BLAST OMCL MP kernels after modified symmetry based updation. The coordinates of each point in the plots are the ROC50 scores for one family, obtained using PSI-BLAST OMCL NM kernel(x-axis) and PSI-BLAST OMCL MP kernel (y-axis).

**Figure 5:**
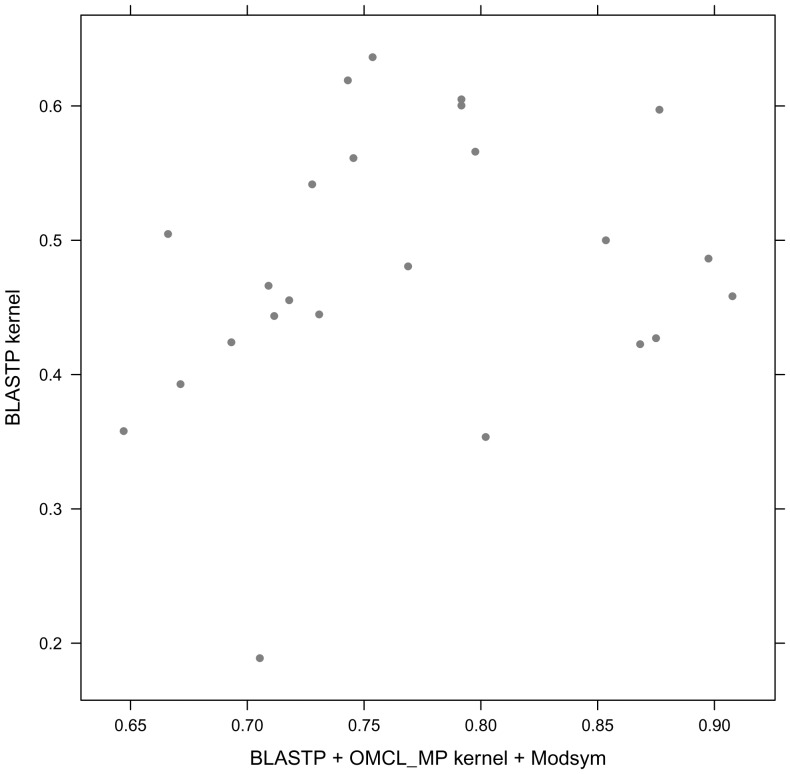
Family-by-family comparison of BLASTP kernel and BLASTP OMCL MP kernel after modified symmetry based updation. The coordinates of each point in the plots are the ROC50 scores for one family, obtained using BLASTP OMCL MP kernel with modified symmetry(x-axis) and BLASTP kernel (y-axis).

Figure 4 shows a family-by-family comparison of the ROC scores of 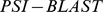 kernel combined with 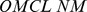 and 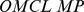 kernels (**VI, VIII**). The points fall approximately near evenly above and below the diagonal, indicating similar performance of both methods. However there exists more points on upper triangle of the Figure 4 which proves a little superiority for 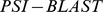 kernel combined with the 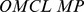 kernel (**VIII**). Figure 5 shows the family distribution for ROC50 scores of 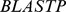 kernel (**I**) and its improvement after combination with the 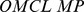 kernel including modified symmetry based enhancements (**XI**). For most of the families, the 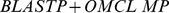 kernel after modified symmetry based reassignment (**XI**) provides higher ROC50 scores than simple 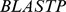 kernel (**I**). All the experiments demonstrate the utility of combined spectral kernel approaches with modified symmetry corrections in the remote homolog detection.

## Discussion

We have presented and experimentally evaluated twelve spectral kernels for remote homology detection that classify protein sequences in comparison with the explicit evaluation of modified symmetry based proximity norm. These kernels measures sequence similarity on the unlabelled data. For this unsupervised protein family classification approach, we focus on our spectral clustering approaches with combined local alignment score-based valid kernels. This approach performs competitively with state-of-the-art neighborhood [22] and profile [23] mismatch kernel methods. When we experiment with introducing modified symmetry in kernel space for homolog detection, our methods outperform earlier known cluster kernel methods in this setting.

Weston et al in [22], [23] introduced the neighborhood and mismatch profile concepts on the BLASTP and PSI-BLAST scores earlier. However, they did not experiment with positive-semidefinitive kernels after singular value decomposition of BLASTP (**I**), PSI-BLAST (**II**) and newly experimented OrthoMCL scores for kernel formations (**III, IV**). After combined with neighborhood similarity and mismatch profile features (**V, VI, VII, VIII**), our proposed Mercer kernels provide significant solutions after introducing modified symmetry based updating (**IX, X, XI, XII**) in spectral clustering results.

Four major observations can be made by analysing different experiments presented in this article. First, the direct use of local-alignment based BLASTP and PSI-BLAST scores to create a kernel matrix with singular value decomposition (**I, II**) proves to be a valid kernel for homology detection. Second, as discussed earlier in coperation of previously detected OrthoMCL scores to reduce the “recent” paralog effects in BLASTP/PSI-BLAST results gains significance. The neighborhood similarity and the mismatch profile kernel over OrthoMCL scores (**III, IV**) also proves to be significant in comparison with earlier cluster kernels, reducing the diagonal dominance issue with arbitrary lower magnitude distribution of diagonal values. Third, we do not need to diagonalize the matrix of all labelled and unlabelled data as in [22]. The leading eigenvectors over the kernel matrix in our spectral clustering implementation. It improves the sensitivity over the all-vs-all local alignment scores for the global distance computation to all proteins without using any hard cut-off threshold. Implicit reduction of inter-cluster edges in spectral clustering also demotes promiscuous domain problem. Without using any relaxation to random walks by restricting to a one-to-one allocations for all proteins among all families it solves this problem, which TribeMCL [20] did with the inflation parameter as a relaxation over the random walks. Four, the modified symmetry based reallocation in kernel space imposed to be biologically significant to exclude outliers as discussed earlier. The intra-symmetrical clusters represent more compact set of homologs based on their similarity scores in the kernel matrix. The nearest neighbors within same cluster represent homologs with similar domains. Smaller distance with the nearest neighbor therefore signifies more compact clusters in kernel space and the nearest neighbors in different clusters represent homologs in different domains. Therefore detecting modified symmetry among multi-domain homolog proteins classifies the protein to a cluster of proteins. The clusters show more accurate domain selection with closer nearest neighbor homologs expressing more biological significance.

Both the widely used cluster kernels [22] and OrthoMCL [19] produce efficient clusters even in the context of remote homolog detection in multi-domain protein families. This fact is reassuring to the validity of our approaches to capture more statistically significant protein clusters with biological relevance of modified symmetry correction.

### Statistical performance evaluation

To evaluate the statistical significance of the differences in the performances observed among all spectral kernels, we perform Wilcoxon signed-rank tests on the area under the ROC50 curve of all simple score-based local alignment kernels, combined spectral kernels and the results after corrections with modified symmetry. Table 4 shows the outputs of this test. Method A outperforms method B according to Wilcoxon test with 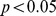. The signed-rank results show expected trends of superiority of position specific scoring, modified symmetry based corrections and the 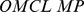 kernel over 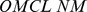 kernel. The median difference values between two methods in Table 4 show the consecutive improvement in cluster results of local alignment kernels after combinations and modified symmetry based updations over them.

**Table 4:** Wilcoxon signed rank test on AUC for ROC50 scores.

ID	Method	ID	Method	Median	p-value
1	1	2	2	Difference	
I	BLASTP kernel	XII	PSI-BLAST +OMCL MP kernel + Modsym	0.808	2.38 e-7
II	PSI-BLAST kernel	III	OMCL NM kernel	0.466	1.19 e-7
III	OMCL NM kernel	X	PSI-BLAST + OMCL NM kernel + Modsym	0.729	1.48 e-2
V	BLASTP + OMCL NM kernel	VIII	PSI-BLAST + OMCL MP kernel	0.483	2.77 e-2
V	BLASTP + OMCL NM kernel	X	PSI-BLAST + OMCL NM kernel + Modsym	0.714	3.45 e-2

OMCL NM  =  OrthoMCL Neighborhood Mismatch kernel, OMCL MP  =  OrthoMCL Mismatch Profile kernel.

### Quantitative performance evaluation

We evaluate the clustering solutions for all kernels objectively by measuring five validity measures Dunn, Davies-Bouldin, Kruskal, Rand and Jaccard indices as defined in [45], [46], [47], [48] and [49] respectively in Table 5. The Dunn validity index [45] shows increasing values for better performance. As a further quantitative evaluation, for the 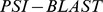 kernel after modified symmetry based corrections and combined with the 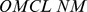 (**X**) and 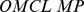 (**XII**) kernels respectively provide Dunn's index values of 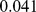 and 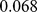 in Table 5. Similarly, the Davies-Bouldin 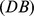 index [46] value shows better clustering solutions with combined 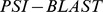 kernel over combined 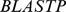 kernel with decreasing values for 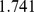 and 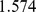 for 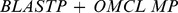 (**VII**) and 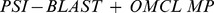 (**VIII**) kernels in Table 5 respectively.

**Table 5:** Performance evaluations on clustering solutions for all kernels.

ID	Kernel	Dunn	DB	Kruskal	Rand	Jaccard
I	BLASTP kernel	0.013	2.174	5.566 e-3	7.951 e-1	2.455 e-2
II	PSI-BLAST kernel	0.015	2.167	5.826 e-3	7.961 e-1	2.577 e-2
III	OMCL NM kernel	0.032	2.159	1.145 e-2	8.020 e-1	2.636 e-2
IV	OMCL MP kernel	0.036	2.157	1.327 e-2	8.026 e-1	2.707 e-2
V	BLASTP + OMCL NM kernel	0.039	2.156	1.418 e-2	8.263 e-1	3.207 e-2
IX	BLASTP + OMCL NM kernel + Modsym	0.039	2.135	1.748 e-2	8.399 e-1	3.295 e-2
VI	PSI-BLAST + OMCL NM kernel	0.040	2.123	1.879 e-2	8.489 e-1	3.824 e-2
X	PSI-BLAST + OMCL NM kernel + Modsym	0.041	1.908	1.999 e-2	8.724 e-1	4.017 e-2
VII	BLASTP + OMCL MP kernel	0.052	1.741	2.253 e-2	8.856 e-1	4.191 e-2
XI	BLASTP + OMCL MP kernel + Modsym	0.053	1.678	3.123 e-2	8.989 e-1	4.205 e-2
VIII	PSI-BLAST + OMCL MP kernel	0.055	1.574	5.097 e-2	8.991 e-1	4.262 e-2
XII	PSI-BLAST + OMCL MP kernel + Modsym	0.068	1.419	6.974 e-2	9.025 e-1	4.289 e-2

OMCL NM  =  OrthoMCL Neighborhood Mismatch kernel, OMCL MP  =  OrthoMCL Mismatch Profile kernel.

The increasing values of 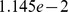 and 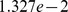 for Kruskal index [47] in Table 5 for 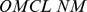 (**III**) and 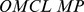 (**IV**) kernels over those values 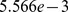 and 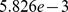 respectively for 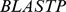 (**I**) and 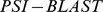 (**II**) kernels, shows the significance of the Markov cluster similarity scoring kernels considering neighborhood similarity and mismatch profile respectively. The Rand index [48] shows the increasing superiority of clustering solutions for 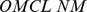 (**III**), 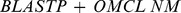 (**V**) and 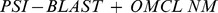 (**VI**) kernels respectively with increasing values of 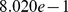, 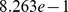 and 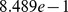 in Table 5 for the quantitative evaluation. The better increasing values of Jaccard index [49] with 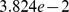, 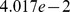, 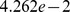 and 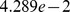 values in Table 5 for 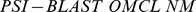 (**VI**), 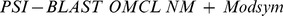 (**X**), 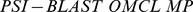 (**VIII**) and 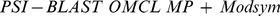 (**XII**) kernels respectively further show the significance of modified symmetry based corrections over the clustering solutions provided by the combined local alignment spectral kernels. This shows superiority of the combined kernels even over local alignment kernels proving 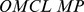 kernel more significant than 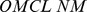 kernel.

### Comparative performance evaluation

We evaluate the clustering solutions of our proposed kernels comparatively with those of the already-existing linear [44], mismatch [22] and string [28] kernels. We experiment those mismatch [22] and string [28] kernels over the BLASTP, PSI-BLAST and OMCL matrices to obtain ROC50 scores provided by those kernels. In Table 6, the ROC50 scores provided by those existing kernels are shown. The ROC50 scores of our proposed kernels in Tables 1, 2, 3, 4 show superior efficiency with higher ROC50 scores. Similarly, to experiment with a large dataset, we run all our proposed kernels as-well-as the state-of-the-art linear [44], string [28] and mismatch [22] kernels on SVM over the existing dataset with 54 families from SCOP version 1.59 [23]. We experiment the existing linear [44] and string [28] kernels over this dataset and compare it with existing results of Spectrum Mismatch kernel [22]. We also experiment our proposed BLASTP, PSI-BLAST, OMCL NM and OMCL MP kernels over this dataset. We also compare the kernel outputs further after the modified symmetry based enhancements. All the ROC50 scores of the clustering solutions provided by all algorithms are included in Table 7. The higher ROC scores provided by our proposed kernels also show superior values over the existing kernels.

**Table 6:** ROC50 averaged over 23 families for different string and mismatch kernels.

Kernel	Dataset matrix	Mean ROC50
Spectrum Mismatch kernel	BLASTP (I)	0.416
	PSI-BLAST (II)	0.430
	OMCL NM (III)	0.465
	OMCL MP (IV)	0.521
String kernel (LIBSVM)	BLASTP (I)	0.495
	PSI-BLAST (II)	0.545
	OMCL NM (III)	0.584
	OMCL MP (IV)	0.550

OMCL NM  =  OrthoMCL Neighborhood Mismatch kernel, OMCL MP  =  OrthoMCL Mismatch Profile kernel.

**Table 7:** ROC50 averaged over existing dataset from SCOP version 1.59 for different string, mismatch and spectral kernels.

Kernel	Mean ROC50
Linear kernel (SPIDER)	0.384
String kernel (LIBSVM)	0.388
Spectrum Mismatch kernel [22]	0.416
BLASTP kernel (I)	0.420
PSI-BLAST kernel (II)	0.433
OMCL NM kernel (III)	0.780
OMCL MP kernel (IV)	0.801
Spectrum Mismatch kernel + Modsym	0.789
BLASTP kernel + Modsym	0.851
PSI-BLAST kernel + Modsym	0.869

OMCL NM  =  OrthoMCL Neighborhood Mismatch kernel, OMCL MP  =  OrthoMCL Mismatch Profile kernel.

## Conclusions

The homologous protein family detection tool within Hemiascomycete yeast complete genomes are appreciated in genomics to detect the conservation of function. Therefore, we propose a computational approach for computing local alignment based Mercer kernels utilizing Markov similarity to reduce “recent” paralog effects. Introducing profile mismatching and neighborhood feature vectors in combined Mercer kernels for spectral clustering, effectively escalates remote homolgy detection from unlabeled protein sequences database. We experiment the corrections by the modified symmetry based proximity norm producing improved clusters with reduced outliers/singletons and selecting more biologically significant domains for multi-domain proteins. Our position specific scoring kernel combined with the modified symmetry based corrections, achieves state-of-the-art prediction performance in the context of unsupervised homology detection. When combined with Markov cluster similarity kernels in well-known neighborhood feature space and considering neighborhood mismatch based on profiles, this approach performs superiorly over other cluster kernels. Therefore to detect the homologs among multi-domain proteins, our spectral clustering approach with combined local alignment kernels results in clusters having better more biological significance. We suggest that this is achieved due to the incorporation of the modified symmetry based corrections in kernel space.

## Supporting Information

Table S1 **List of 23 multidomain family names used from Genolevures database.**
(TXT)

Table S2 **PSI-BLAST kernel matrix.**
(TXT)

Table S3
**OrthoMCL Neighborhood Mismatch** 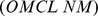 **kernel matrix.**
(TXT)

Table S4
**OrthoMCL Mismatch Profile** 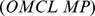 **kernel matrix.**
(TXT)

Table S5
**Combined** 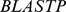 **+** 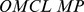 **kernel matrix.**
(TXT)

Table S6
**Combined** 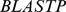 **+** 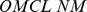 **kernel matrix.**
(TXT)

Table S7
**Combined** 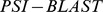 **+** 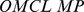 **kernel matrix.**
(TXT)

Table S8
**Combined** 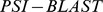 **+** 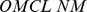 **kernel matrix.**
(TXT)

Table S9 **ROC50 scores obtained over all families.**
(PDF)

Table S10 **ROC scores obtained over all families.**
(PDF)

Table S11 **ROC50 scores obtained after modified symmetry based correction over all families.**
(CSV)

Table S12 **ROC scores obtained after modified symmetry based correction over all families.**
(CSV)
