# Supplementary material for: Searching Remote Homology with Spectral Clustering with Symmetry in Neighborhood Cluster Kernels
Source: PLoS One. 2013 Feb 15;8(2):e46468. doi: 10.1371/journal.pone.0046468 (PMC3574063; doi:10.1371/journal.pone.0046468)
Supplement: Table S9 — ROC50 scores obtained over all families. (PDF) [file pone.0046468.s009.pdf]

Family	Kernel				
	blastp_kernel	psimatrixkernel	omclNNkernel	omclmismatchprofilekernel	dot_psi_omcl_NN_kernel
ROC50-fam1	0.5000000	0.4744409	0.7207792	0.6509740	0.7013889
ROC50-fam2	0.4448052	0.4776182	0.7184066	0.6899350	0.5925000
ROC50-fam3	0.4661654	0.5385309	0.7202381	0.8174603	0.6428571
ROC50-fam4	0.5046992	0.5386704	0.7482304	0.8971088	0.6022222
ROC50-fam5	0.3928571	0.4668770	0.8142857	0.8174603	0.8750000
ROC50-fam6	0.6003401	0.5028802	0.7420635	0.8035714	0.7314050
ROC50-fam7	0.5416667	0.5079770	0.6750000	0.8916667	0.6887255
ROC50-fam8	0.6190476	0.4761146	0.7000000	0.9050000	0.8376623
ROC50-fam9	0.5659722	0.5527337	0.7986111	0.5896057	0.9230770
ROC50-fam10	0.5972222	0.5434822	0.6626984	0.6626984	0.5952381
ROC50-fam11	0.4553571	0.4588608	0.7291667	0.7708333	0.9545455
ROC50-fam12	0.4435897	0.4926645	0.7041667	0.6809524	0.7159091
ROC50-fam13	0.4583333	0.4391026	0.6262626	0.8458333	0.8181818
ROC50-fam14	0.6363636	0.4554140	0.8346154	0.7100000	0.8500000
ROC50-fam15	0.6049107	0.4661290	0.7142857	0.8653846	0.7047397
ROC50-fam16	0.4270833	0.4615385	0.7916667	0.7310606	0.6875000
ROC50-fam17	0.4226190	0.4857105	0.7731481	0.8846154	0.8675214
ROC50-fam18	0.4863636	0.5544583	0.7000000	0.6285266	0.9086538
ROC50-fam19	0.3579545	0.5391614	0.8303571	0.6375000	0.7888889
ROC50-fam20	0.1888889	0.4825397	0.7986111	0.7905983	0.9230770
ROC50-fam21	0.5612245	0.4747634	0.8660714	0.7785714	0.6956522
ROC50-fam22	0.4240260	0.5266436	0.6166667	0.7168067	0.5833333
ROC50-fam23	0.3535354	0.4788489	0.7685185	0.6388889	0.7297297
MEAN ROC50	0.4805663	0.4954418	0.7414717	0.7567414	0.7572960

dot_psi_omcl_MP_kernel	dot_blastp_omcl_NN_kernel	dot_blastp_omcl_MP_kernel	dot_blastp_omcl_NN_MODSYM	dot_blastp_omcl_MP_MODSYM	dot_psi_omcl_NN_MODSYM
0.8318182	0.8776224	0.7513369	0.6636364	0.8535354	0.6818182
0.6899350	0.6250000	0.6646825	0.6398810	0.7307692	0.9135714
0.7785714	0.8095238	0.6785714	0.7202381	0.7090909	0.7571429
0.8298872	0.6899350	0.6646825	0.6250000	0.6660839	0.6540179
0.5357143	0.7000000	0.7202381	0.8174603	0.6714286	0.8660714
0.7455357	0.7455357	0.9311224	0.7076720	0.7916667	0.9642857
0.8560000	0.8175926	0.6906250	0.7673077	0.7277778	0.6750000
0.8291667	0.8346154	0.8600000	0.7357143	0.7430556	0.8600000
0.8131313	0.7539683	0.7091503	0.7905983	0.7976190	0.9444444
0.8944444	0.7939815	0.7449495	0.6388889	0.8764205	0.7222222
0.6597222	0.6562500	0.6156250	0.6458333	0.7179487	0.8281250
0.7848485	0.6555556	0.9047619	0.7551282	0.7115385	0.7333333
0.7731481	0.6891026	0.6278409	0.8277778	0.9076923	0.7760417
0.8611111	0.7533333	0.8346154	0.7785714	0.7536765	0.6185345
0.8504464	0.7142857	0.7670068	0.6887755	0.7916667	0.9642857
0.7440476	0.7083333	0.7395833	0.8846154	0.8750000	0.8083333
0.8458333	0.8869048	0.9198718	0.8446970	0.8681818	0.8083333
0.6893940	0.8719008	0.8776224	0.7207792	0.8974359	0.7045455
0.8819444	0.7767857	0.8011364	0.6562500	0.6470588	0.8375000
0.7539683	0.6851852	0.6944444	0.6481481	0.7053571	0.6694444
0.6344538	0.6428571	0.6479592	0.8174603	0.7454545	0.6428571
0.6714286	0.6357143	0.6523810	0.7066234	0.6931818	0.9718894
0.8425926	0.6712963	0.8174603	0.9722222	0.8020833	0.9722222
0.7737888	0.7389252	0.7528551	0.7414469	0.7688575	0.7988704

dot_psi_omcl_MP_MODSYM
0.7000000
0.7992203
0.7179487
0.8148148
0.9166667
0.7102564
0.9497608
0.5962963
0.7708333
0.7205882
0.7619048
0.9642857
0.7121212
0.5291902
0.6948718
0.7878788
0.9545455
0.7250000
0.9285714
0.9375000
0.7500000
0.7352941
0.9705882
0.7890494
