# Supplementary material for: Searching Remote Homology with Spectral Clustering with Symmetry in Neighborhood Cluster Kernels
Source: PLoS One. 2013 Feb 15;8(2):e46468. doi: 10.1371/journal.pone.0046468 (PMC3574063; doi:10.1371/journal.pone.0046468)
Supplement: Table S10 — ROC scores obtained over all families. (PDF) [file pone.0046468.s010.pdf]

Family	Kernel			
	blastp_kernel	psimatrixkernel	omclNNkernel	omclmismatchprofilekernel
ROC-fam1	0.7692308	0.9356261	0.9375000	0.9827586
ROC-fam2	0.8207070	0.9767682	0.9656085	0.9598765
ROC-fam3	0.9772727	0.8912226	0.9615385	0.9500000
ROC-fam4	0.7757576	0.9765100	0.9978505	0.9806397
ROC-fam5	0.8636364	0.8940439	0.9444444	0.9500000
ROC-fam6	0.8125000	0.9524573	0.9500000	0.9580420
ROC-fam7	0.8000000	0.9685004	0.9254386	0.9800000
ROC-fam8	0.7692308	0.9303797	0.9285714	0.9595960
ROC-fam9	0.9027778	0.9220370	0.9444444	0.9843750
ROC-fam10	0.7812500	0.9647930	0.9375000	0.9375000
ROC-fam11	0.8846154	0.9101153	0.9285714	0.9615385
ROC-fam12	0.8500000	0.9568390	0.9444444	0.9375000
ROC-fam13	0.9117647	0.9455414	0.9866310	0.9586777
ROC-fam14	0.8846154	0.9301820	0.9642857	0.9259260
ROC-fam15	0.8000000	0.9533920	0.9444444	0.9642857
ROC-fam16	0.8500000	0.9444268	0.9736842	0.9772727
ROC-fam17	0.9032258	0.9458599	0.9500000	0.9675325
ROC-fam18	0.8250000	0.9396825	0.9250000	0.9850000
ROC-fam19	0.8235294	0.9109015	0.9464286	0.9761905
ROC-fam20	0.7000000	0.9233890	0.9444444	0.9642857
ROC-fam21	0.8235294	0.8949843	0.9444444	0.9545455
ROC-fam22	0.8627874	0.9803187	0.9285714	0.9736842
ROC-fam23	0.8500000	0.9644886	0.9615385	0.9000000
MEAN ROC	0.8365839	0.9396721	0.9493646	0.9604012

dot_psi_omcl_NN_kernel	dot_psi_omcl_MP_kernel	dot_blastp_omcl_NN_kernel	dot_blastp_omcl_MP_kernel
0.9285714	0.9545455	0.9642857	0.9750000
0.9166667	0.9598765	0.9166667	0.9518519
0.9839744	0.9545455	0.9404762	0.9666667
0.9285714	0.9768519	0.9598765	0.9518519
0.9500000	0.9947090	0.9166667	0.9615385
0.9444444	0.9487180	0.9487180	0.9692308
0.9500000	0.9817814	0.9840226	0.9473684
0.9444444	0.9658120	0.9642857	0.9545455
0.9545455	0.9635417	0.9375000	0.9722222
0.9000000	0.9724265	0.9638010	0.9583333
0.9444444	0.9736842	0.9000000	0.9795918
0.9444444	0.9583333	0.8750000	0.9690476
0.9494950	0.9500000	0.9814815	0.9919192
0.9513889	0.9555556	0.9722222	0.9642857
0.9976589	0.9751131	0.9444444	0.9807692
0.9242424	0.9431818	0.9375000	0.9444444
0.9545455	0.9586777	0.9666667	0.9642857
0.9545455	0.9285714	0.9583333	0.9642857
0.9285714	0.9500000	0.9375000	0.9583333
0.9545455	0.9375000	0.8906250	0.9736842
0.9761905	0.9722222	0.8750000	0.9722222
0.8750000	0.9626697	0.9460784	0.9348740
0.9873950	0.9687500	0.9974425	0.9666667
0.9453776	0.9611768	0.9425475	0.9640443

dot_blastp_omcl_NN_MODSYM	dot_blastp_omcl_MP_MODSYM	dot_psi_omcl_NN_MODSYM	dot_psi_omcl_MP_MODSYM
0.9498983	0.9916630	0.9968454	0.9977072
0.9752051	0.9864583	0.9903472	0.9978059
0.9206850	0.9932503	0.9953271	0.9884013
0.9761100	0.9900000	0.9891667	0.9980640
0.9254169	0.9984424	0.9922118	0.9921630
0.9419355	0.7984076	0.8984076	0.9861111
0.9661184	0.9922890	0.9907670	0.9950980
0.9452230	0.9952830	0.9921384	0.9960443
0.9380952	0.9927900	0.7984326	0.9903110
0.9678650	0.9947005	0.9626728	0.9668222
0.9347310	0.9957031	0.9914062	0.8312369
0.9488673	0.8620390	0.9905606	0.9875093
0.9520566	0.9914953	0.9912975	0.9914013
0.9461783	0.9984277	0.8301887	0.9960443
0.9580645	0.7984076	0.8984076	0.9967949
0.9552618	0.9920886	0.9905063	0.9926752
0.9543270	0.9924842	0.9905063	0.9939490
0.9493175	0.9905363	0.9269941	0.9962963
0.9339399	0.9937500	0.9953125	0.9143082
0.9416226	0.9968652	0.9858934	0.8551150
0.9254169	0.9906542	0.9984424	0.9921630
0.8794859	0.9839810	0.9951007	0.9749557
0.9722222	0.8555300	0.9269585	0.8733766
0.9460019	0.9641411	0.9616475	0.9697545
